# Hospitalization Rates and Characteristics of Patients Hospitalized with Laboratory-Confirmed Coronavirus Disease 2019 — COVID-NET, 14 States, March 1–30, 2020

**DOI:** 10.15585/mmwr.mm6915e3

**Published:** 2020-04-17

**Authors:** Shikha Garg, Lindsay Kim, Michael Whitaker, Alissa O’Halloran, Charisse Cummings, Rachel Holstein, Mila Prill, Shua J. Chai, Pam D. Kirley, Nisha B. Alden, Breanna Kawasaki, Kimberly Yousey-Hindes, Linda Niccolai, Evan J. Anderson, Kyle P. Openo, Andrew Weigel, Maya L. Monroe, Patricia Ryan, Justin Henderson, Sue Kim, Kathy Como-Sabetti, Ruth Lynfield, Daniel Sosin, Salina Torres, Alison Muse, Nancy M. Bennett, Laurie Billing, Melissa Sutton, Nicole West, William Schaffner, H. Keipp Talbot, Clarissa Aquino, Andrea George, Alicia Budd, Lynnette Brammer, Gayle Langley, Aron J. Hall, Alicia Fry

**Affiliations:** ^1^CDC COVID-NET Team; ^2^Eagle Global Scientific, Atlanta, Georgia; ^3^Chickasaw Nation Industries, Norman, Oklahoma; ^4^Oak Ridge Institute for Science and Education, Oak Ridge, Tennessee; ^5^California Emerging Infections Program, Oakland, California; ^6^Communicable Disease Branch, Colorado Department of Public Health and Environment, Denver, Colorado; ^7^Connecticut Emerging Infections Program, Yale School of Public Health, New Haven, Connecticut; ^8^Departments of Medicine and Pediatrics, Emory University School of Medicine, Atlanta, Georgia; ^9^Emerging Infections Program, Georgia Department of Health, Atlanta, Georgia; ^10^Veterans Affairs Medical Center, Atlanta, Georgia; ^11^Foundation for Atlanta Veterans Education and Research, Decatur, Georgia; ^12^Iowa Department of Public Health; ^13^Maryland Department of Health; ^14^Communicable Disease Division, Michigan Department of Health and Human Services, Lansing, Michigan; ^15^Minnesota Department of Health; ^16^New Mexico Department of Health; ^17^New York State Department of Health; ^18^University of Rochester School of Medicine and Dentistry, Rochester, New York; ^19^Bureau of Infectious Diseases, Ohio Department of Health, Columbus, Ohio; ^20^Oregon Public Health Division; ^21^Division of Infectious Disease, Vanderbilt University School of Medicine, Nashville, Tennessee; ^22^Salt Lake County Health Department, Salt Lake City, Utah.

Since SARS-CoV-2, the novel coronavirus that causes coronavirus disease 2019 (COVID-19), was first detected in December 2019 (*1*), approximately 1.3 million cases have been reported worldwide (*2*), including approximately 330,000 in the United States (*3*). To conduct population-based surveillance for laboratory-confirmed COVID-19–associated hospitalizations in the United States, the COVID-19–Associated Hospitalization Surveillance Network (COVID-NET) was created using the existing infrastructure of the Influenza Hospitalization Surveillance Network (FluSurv-NET) (*4*) and the Respiratory Syncytial Virus Hospitalization Surveillance Network (RSV-NET). This report presents age-stratified COVID-19–associated hospitalization rates for patients admitted during March 1–28, 2020, and clinical data on patients admitted during March 1–30, 2020, the first month of U.S. surveillance. Among 1,482 patients hospitalized with COVID-19, 74.5% were aged ≥50 years, and 54.4% were male. The hospitalization rate among patients identified through COVID-NET during this 4-week period was 4.6 per 100,000 population. Rates were highest (13.8) among adults aged ≥65 years. Among 178 (12%) adult patients with data on underlying conditions as of March 30, 2020, 89.3% had one or more underlying conditions; the most common were hypertension (49.7%), obesity (48.3%), chronic lung disease (34.6%), diabetes mellitus (28.3%), and cardiovascular disease (27.8%). These findings suggest that older adults have elevated rates of COVID-19–associated hospitalization and the majority of persons hospitalized with COVID-19 have underlying medical conditions. These findings underscore the importance of preventive measures (e.g., social distancing, respiratory hygiene, and wearing face coverings in public settings where social distancing measures are difficult to maintain)^†^ to protect older adults and persons with underlying medical conditions, as well as the general public. In addition, older adults and persons with serious underlying medical conditions should avoid contact with persons who are ill and immediately contact their health care provider(s) if they have symptoms consistent with COVID-19 (https://www.cdc.gov/coronavirus/2019-ncov/symptoms-testing/symptoms.html) (*5*). Ongoing monitoring of hospitalization rates, clinical characteristics, and outcomes of hospitalized patients will be important to better understand the evolving epidemiology of COVID-19 in the United States and the clinical spectrum of disease, and to help guide planning and prioritization of health care system resources.

COVID-NET conducts population-based surveillance for laboratory-confirmed COVID-19–associated hospitalizations among persons of all ages in 99 counties in 14 states (California, Colorado, Connecticut, Georgia, Iowa, Maryland, Michigan, Minnesota, New Mexico, New York, Ohio, Oregon, Tennessee, and Utah), distributed across all 10 U.S Department of Health and Human Services regions.^§^ The catchment area represents approximately 10% of the U.S. population. Patients must be residents of a designated COVID-NET catchment area and hospitalized within 14 days of a positive SARS-CoV-2 test to meet the surveillance case definition. Testing is requested at the discretion of treating health care providers. Laboratory-confirmed SARS-CoV-2 is defined as a positive result by any test that has received Emergency Use Authorization for SARS-CoV-2 testing.^¶^ COVID-NET surveillance officers in each state identify cases through active review of notifiable disease and laboratory databases and hospital admission and infection control practitioner logs. Weekly age-stratified hospitalization rates are estimated using the number of catchment area residents hospitalized with laboratory-confirmed COVID-19 as the numerator and National Center for Health Statistics vintage 2018 bridged-race postcensal population estimates for the denominator.** As of April 3, 2020, COVID-NET hospitalization rates are being published each week at https://gis.cdc.gov/grasp/covidnet/COVID19_3.html. For each case, trained surveillance officers conduct medical chart abstractions using a standard case report form to collect data on patient characteristics, underlying medical conditions, clinical course, and outcomes. Chart reviews are finalized once patients have a discharge disposition. COVID-NET surveillance was initiated on March 23, 2020, with retrospective case identification of patients admitted during March 1–22, 2020, and prospective case identification during March 23–30, 2020. Clinical data on underlying conditions and symptoms at admission are presented through March 30; hospitalization rates are updated weekly and, therefore, are presented through March 28 (epidemiologic week 13).

The COVID-19–associated hospitalization rate among patients identified through COVID-NET for the 4-week period ending March 28, 2020, was 4.6 per 100,000 population (Figure 1). Hospitalization rates increased with age, with a rate of 0.3 in persons aged 0–4 years, 0.1 in those aged 5–17 years, 2.5 in those aged 18–49 years, 7.4 in those aged 50–64 years, and 13.8 in those aged ≥65 years. Rates were highest among persons aged ≥65 years, ranging from 12.2 in those aged 65–74 years to 17.2 in those aged ≥85 years. More than half (805; 54.4%) of hospitalizations occurred among men; COVID-19-associated hospitalization rates were higher among males than among females (5.1 versus 4.1 per 100,000 population). Among the 1,482 laboratory-confirmed COVID-19–associated hospitalizations reported through COVID-NET, six (0.4%) each were patients aged 0–4 years and 5–17 years, 366 (24.7%) were aged 18–49 years, 461 (31.1%) were aged 50–64 years, and 643 (43.4%) were aged ≥65 years. Among patients with race/ethnicity data (580), 261 (45.0%) were non-Hispanic white (white), 192 (33.1%) were non-Hispanic black (black), 47 (8.1%) were Hispanic, 32 (5.5%) were Asian, two (0.3%) were American Indian/Alaskan Native, and 46 (7.9%) were of other or unknown race. Rates varied widely by COVID-NET surveillance site (Figure 2).

**FIGURE 1:**
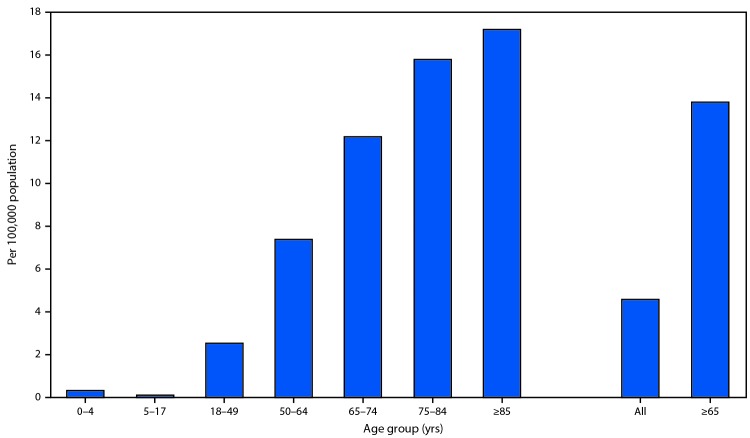
Laboratory-confirmed coronavirus disease 2019 (COVID-19)–associated hospitalization rates,* by age group — COVID-NET, 14 states,^†^ March 1–28, 2020 **Abbreviation:** COVID-NET = Coronavirus Disease 2019–Associated Hospitalization Surveillance Network. * Number of patients hospitalized with COVID-19 per 100,000 population. ^†^ Counties included in COVID-NET surveillance: California (Alameda, Contra Costa, and San Francisco counties); Colorado (Adams, Arapahoe, Denver, Douglas, and Jefferson counties); Connecticut (New Haven and Middlesex counties); Georgia (Clayton, Cobb, DeKalb, Douglas, Fulton, Gwinnett, Newton, and Rockdale counties); Iowa (one county represented); Maryland (Allegany, Anne Arundel, Baltimore, Baltimore City, Calvert, Caroline, Carroll, Cecil, Charles, Dorchester, Frederick, Garrett, Harford, Howard, Kent, Montgomery, Prince George’s, Queen Anne’s, St. Mary’s, Somerset, Talbot, Washington, Wicomico, and Worcester counties); Michigan (Clinton, Eaton, Genesee, Ingham, and Washtenaw counties); Minnesota (Anoka, Carver, Dakota, Hennepin, Ramsey, Scott, and Washington counties); New Mexico (Bernalillo, Chaves, Dona Ana, Grant, Luna, San Juan, and Santa Fe counties); New York (Albany, Columbia, Genesee, Greene, Livingston, Monroe, Montgomery, Ontario, Orleans, Rensselaer, Saratoga, Schenectady, Schoharie, Wayne, and Yates counties); Ohio (Delaware, Fairfield, Franklin, Hocking, Licking, Madison, Morrow, Perry, Pickaway and Union counties); Oregon (Clackamas, Multnomah, and Washington counties); Tennessee (Cheatham, Davidson, Dickson, Robertson, Rutherford, Sumner, Williamson, and Wilson counties); and Utah (Salt Lake County).

**FIGURE 2:**
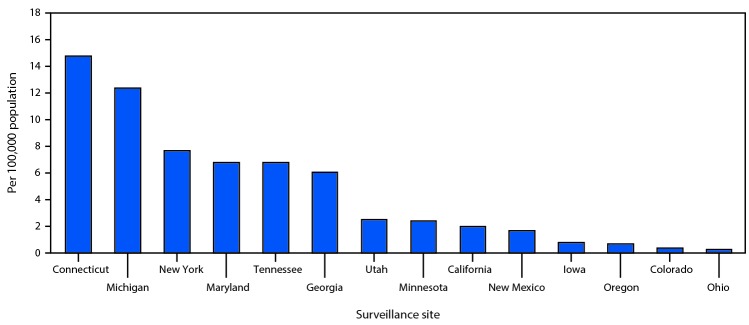
Laboratory-confirmed coronavirus disease 2019 (COVID-19)–associated hospitalization rates,* by surveillance site^†^— COVID-NET, 14 states, March 1–28, 2020 **Abbreviation:** COVID-NET = Coronavirus Disease 2019–Associated Hospitalization Surveillance Network. * Number of patients hospitalized with COVID-19 per 100,000 population. ^†^ Counties included in COVID-NET surveillance: California (Alameda, Contra Costa, and San Francisco counties); Colorado (Adams, Arapahoe, Denver, Douglas, and Jefferson counties); Connecticut (New Haven and Middlesex counties); Georgia (Clayton, Cobb, DeKalb, Douglas, Fulton, Gwinnett, Newton, and Rockdale counties); Iowa (one county represented); Maryland (Allegany, Anne Arundel, Baltimore, Baltimore City, Calvert, Caroline, Carroll, Cecil, Charles, Dorchester, Frederick, Garrett, Harford, Howard, Kent, Montgomery, Prince George’s, Queen Anne’s, St. Mary’s, Somerset, Talbot, Washington, Wicomico, and Worcester counties); Michigan (Clinton, Eaton, Genesee, Ingham, and Washtenaw counties); Minnesota (Anoka, Carver, Dakota, Hennepin, Ramsey, Scott, and Washington counties); New Mexico (Bernalillo, Chaves, Dona Ana, Grant, Luna, San Juan, and Santa Fe counties); New York (Albany, Columbia, Genesee, Greene, Livingston, Monroe, Montgomery, Ontario, Orleans, Rensselaer, Saratoga, Schenectady, Schoharie, Wayne, and Yates counties); Ohio (Delaware, Fairfield, Franklin, Hocking, Licking, Madison, Morrow, Perry, Pickaway and Union counties); Oregon (Clackamas, Multnomah, and Washington counties); Tennessee (Cheatham, Davidson, Dickson, Robertson, Rutherford, Sumner, Williamson, and Wilson counties); and Utah (Salt Lake County).

During March 1–30, underlying medical conditions and symptoms at admission were reported through COVID-NET for approximately 180 (12.1%) hospitalized adults (Table); 89.3% had one or more underlying conditions. The most commonly reported were hypertension (49.7%), obesity (48.3%), chronic lung disease (34.6%), diabetes mellitus (28.3%), and cardiovascular disease (27.8%). Among patients aged 18–49 years, obesity was the most prevalent underlying condition, followed by chronic lung disease (primarily asthma) and diabetes mellitus. Among patients aged 50–64 years, obesity was most prevalent, followed by hypertension and diabetes mellitus; and among those aged ≥65 years, hypertension was most prevalent, followed by cardiovascular disease and diabetes mellitus. Among 33 females aged 15–49 years hospitalized with COVID-19, three (9.1%) were pregnant. Among 167 patients with available data, the median interval from symptom onset to admission was 7 days (interquartile range [IQR] = 3–9 days). The most common signs and symptoms at admission included cough (86.1%), fever or chills (85.0%), and shortness of breath (80.0%). Gastrointestinal symptoms were also common; 26.7% had diarrhea, and 24.4% had nausea or vomiting.

**TABLE:** Underlying conditions and symptoms among adults aged ≥18 years with coronavirus disease 2019 (COVID-19)–associated hospitalizations — COVID-NET, 14 states,* March 1–30, 2020^†^

Underlying condition	Age group (yrs), no./total no. (%)			
	Overall	18–49	50–64	≥65 years
**Any underlying condition**	**159/178 (89.3)**	**41/48 (85.4)**	**51/59 (86.4)**	**67/71 (94.4)**
Hypertension	79/159 (49.7)	7/40 (17.5)	27/57 (47.4)	45/62 (72.6)
Obesity^§^	73/151 (48.3)	23/39 (59.0)	25/51 (49.0)	25/61 (41.0)
Chronic metabolic disease^¶^	60/166 (36.1)	10/46 (21.7)	21/56 (37.5)	29/64 (45.3)
Diabetes mellitus	47/166 (28.3)	9/46 (19.6)	18/56 (32.1)	20/64 (31.3)
Chronic lung disease	55/159 (34.6)	16/44 (36.4)	15/53 (28.3)	24/62 (38.7)
Asthma	27/159 (17.0)	12/44 (27.3)	7/53 (13.2)	8/62 (12.9)
Chronic obstructive pulmonary disease	17/159 (10.7)	0/44 (0.0)	3/53 (5.7)	14/62 (22.6)
Cardiovascular disease**	45/162 (27.8)	2/43 (4.7)	11/56 (19.6)	32/63 (50.8)
Coronary artery disease	23/162 (14.2)	0/43 (0.0)	7/56 (12.5)	16/63 (25.4)
Congestive heart failure	11/162 (6.8)	2/43 (4.7)	3/56 (5.4)	6/63 (9.5)
Neurologic disease	22/157 (14.0)	4/42 (9.5)	4/55 (7.3)	14/60 (23.3)
Renal disease	20/153 (13.1)	3/41 (7.3)	2/53 (3.8)	15/59 (25.4)
Immunosuppressive condition	15/156 (9.6)	5/43 (11.6)	4/54 (7.4)	6/59 (10.2)
Gastrointestinal/Liver disease	10/152 (6.6)	4/42 (9.5)	0/54 (0.0)	6/56 (10.7)
Blood disorder	9/156 (5.8)	1/43 (2.3)	1/55 (1.8)	7/58 (12.1)
Rheumatologic/Autoimmune disease	3/154 (1.9)	1/42 (2.4)	0/54 (0.0)	2/58 (3.4)
Pregnancy^††^	3/33 (9.1)	3/33 (9.1)	N/A	N/A
**Symptom** ^§§^				
Cough	155/180 (86.1)	43/47 (91.5)	54/60 (90.0)	58/73 (79.5)
Fever/Chills	153/180 (85.0)	38/47 (80.9)	53/60 (88.3)	62/73 (84.9)
Shortness of breath	144/180 (80.0)	40/47 (85.1)	50/60 (83.3)	54/73 (74.0)
Myalgia	62/180 (34.4)	20/47 (42.6)	23/60 (38.3)	19/73 (26.0)
Diarrhea	48/180 (26.7)	10/47 (21.3)	17/60 (28.3)	21/73 (28.8)
Nausea/Vomiting	44/180 (24.4)	12/47 (25.5)	17/60 (28.3)	15/73 (20.5)
Sore throat	32/180 (17.8)	8/47 (17.0)	13/60 (21.7)	11/73 (15.1)
Headache	29/180 (16.1)	10/47 (21.3)	12/60 (20.0)	7/73 (9.6)
Nasal congestion/Rhinorrhea	29/180 (16.1)	8/47 (17.0)	13/60 (21.7)	8/73 (11.0)
Chest pain	27/180 (15.0)	9/47 (19.1)	13/60 (21.7)	5/73 (6.8)
Abdominal pain	15/180 (8.3)	6/47 (12.8)	6/60 (10.0)	3/73 (4.1)
Wheezing	12/180 (6.7)	3/47 (6.4)	2/60 (3.3)	7/73 (9.6)
Altered mental status/Confusion	11/180 (6.1)	3/47 (6.4)	2/60 (3.3)	6/73 (8.2)

**Abbreviations:** COVID-NET = Coronavirus Disease 2019–Associated Hospitalization Surveillance Network; N/A = not applicable.

* Counties included in COVID-NET surveillance: California (Alameda, Contra Costa, and San Francisco counties); Colorado (Adams, Arapahoe, Denver, Douglas, and Jefferson counties); Connecticut (New Haven and Middlesex counties); Georgia (Clayton, Cobb, DeKalb, Douglas, Fulton, Gwinnett, Newton, and Rockdale counties); Iowa (one county represented); Maryland (Allegany, Anne Arundel, Baltimore, Baltimore City, Calvert, Caroline, Carroll, Cecil, Charles, Dorchester, Frederick, Garrett, Harford, Howard, Kent, Montgomery, Prince George’s, Queen Anne’s, St. Mary’s, Somerset, Talbot, Washington, Wicomico, and Worcester counties); Michigan (Clinton, Eaton, Genesee, Ingham, and Washtenaw counties); Minnesota (Anoka, Carver, Dakota, Hennepin, Ramsey, Scott, and Washington counties); New Mexico (Bernalillo, Chaves, Dona Ana, Grant, Luna, San Juan, and Santa Fe counties); New York (Albany, Columbia, Genesee, Greene, Livingston, Monroe, Montgomery, Ontario, Orleans, Rensselaer, Saratoga, Schenectady, Schoharie, Wayne, and Yates counties); Ohio (Delaware, Fairfield, Franklin, Hocking, Licking, Madison, Morrow, Perry, Pickaway and Union counties); Oregon (Clackamas, Multnomah, and Washington counties); Tennessee (Cheatham, Davidson, Dickson, Robertson, Rutherford, Sumner, Williamson, and Wilson counties); and Utah (Salt Lake County).

^†^ COVID-NET included data for one child aged 5–17 years with underlying medical conditions and symptoms at admission; data for this child are not included in this table. This child was reported to have chronic lung disease (asthma). Symptoms included fever, cough, gastrointestinal symptoms, shortness of breath, chest pain, and a sore throat on admission.

^§^ Obesity is defined as calculated body mass index (BMI) ≥30 kg/m^2^, and if BMI is missing, by *International Classification of Diseases* discharge diagnosis codes. Among 73 patients with obesity, 51 (69.9%) had obesity defined as BMI 30–<40 kg/m^2^, and 22 (30.1%) had severe obesity defined as BMI ≥40 kg/m^2^.

^¶^ Among the 60 patients with chronic metabolic disease, 45 had diabetes mellitus only, 13 had thyroid dysfunction only, and two had diabetes mellitus and thyroid dysfunction.

** Cardiovascular disease excludes hypertension.

^††^ Restricted to women aged 15–49 years.

^§§^ Symptoms were collected through review of admission history and physical exam notes in the medical record and might be determined by subjective or objective findings. In addition to the symptoms in the table, the following less commonly reported symptoms were also noted for adults with information on symptoms (180): hemoptysis/bloody sputum (2.2%), rash (1.1%), conjunctivitis (0.6%), and seizure (0.6%).

## Discussion

During March 1–28, 2020, the overall laboratory-confirmed COVID-19–associated hospitalization rate was 4.6 per 100,000 population; rates increased with age, with the highest rates among adults aged ≥65 years. Approximately 90% of hospitalized patients identified through COVID-NET had one or more underlying conditions, the most common being obesity, hypertension, chronic lung disease, diabetes mellitus, and cardiovascular disease.

Using the existing infrastructure of two respiratory virus surveillance platforms, COVID-NET was implemented to produce robust, weekly, age-stratified hospitalization rates using standardized data collection methods. These data are being used, along with data from other surveillance platforms (https://www.cdc.gov/coronavirus/2019-ncov/covid-data/covidview.html), to monitor COVID-19 disease activity and severity in the United States. During the first month of surveillance, COVID-NET hospitalization rates ranged from 0.1 per 100,000 population in persons aged 5–17 years to 17.2 per 100,000 population in adults aged ≥85 years, whereas cumulative influenza hospitalization rates during the first 4 weeks of each influenza season (epidemiologic weeks 40–43) over the past 5 seasons have ranged from 0.1 in persons aged 5–17 years to 2.2–5.4 in adults aged ≥85 years (*6*). COVID-NET rates during this first 4-week period of surveillance are preliminary and should be interpreted with caution; given the rapidly evolving nature of the COVID-19 pandemic, rates are expected to increase as additional cases are identified and as SARS-CoV-2 testing capacity in the United States increases.

In the COVID-NET catchment population, approximately 49% of residents are male and 51% of residents are female, whereas 54% of COVID-19-associated hospitalizations occurred in males and 46% occurred in females. These data suggest that males may be disproportionately affected by COVID-19 compared with females. Similarly, in the COVID-NET catchment population, approximately 59% of residents are white, 18% are black, and 14% are Hispanic; however, among 580 hospitalized COVID-19 patients with race/ethnicity data, approximately 45% were white, 33% were black, and 8% were Hispanic, suggesting that black populations might be disproportionately affected by COVID-19. These findings, including the potential impact of both sex and race on COVID-19-associated hospitalization rates, need to be confirmed with additional data.

Most of the hospitalized patients had underlying conditions, some of which are recognized to be associated with severe COVID-19 disease, including chronic lung disease, cardiovascular disease, diabetes mellitus (*5*). COVID-NET does not collect data on nonhospitalized patients; thus, it was not possible to compare the prevalence of underlying conditions in hospitalized versus nonhospitalized patients. Many of the documented underlying conditions among hospitalized COVID-19 patients are highly prevalent in the United States. According to data from the National Health and Nutrition Examination Survey, hypertension prevalence among U.S. adults is 29% overall, ranging from 7.5%–63% across age groups (*7*), and age-adjusted obesity prevalence is 42% (range across age groups = 40%–43%) (*8*). Among hospitalized COVID-19 patients, hypertension prevalence was 50% (range across age groups = 18%–73%), and obesity prevalence was 48% (range across age groups = 41%–59%). In addition, the prevalences of several underlying conditions identified through COVID-NET were similar to those for hospitalized influenza patients identified through FluSurv-NET during influenza seasons 2014–15 through 2018–19: 41%–51% of patients had cardiovascular disease (excluding hypertension), 39%–45% had chronic metabolic disease, 33%–40% had obesity, and 29%–31% had chronic lung disease (*6*). Data on hypertension are not collected by FluSurv-NET. Among women aged 15–49 years hospitalized with COVID-19 and identified through COVID-NET, 9% were pregnant, which is similar to an estimated 9.9% of the general population of women aged 15–44 years who are pregnant at any given time based on 2010 data.^††^ Similar to other reports from the United States (*9*) and China (*1*), these findings indicate that a high proportion of U.S. patients hospitalized with COVID-19 are older and have underlying medical conditions.

The findings in this report are subject to at least three limitations. First, hospitalization rates by age and COVID-NET site are preliminary and might change as additional cases are identified from this surveillance period. Second, whereas minimum case data to produce weekly age-stratified hospitalization rates are usually available within 7 days of case identification, availability of detailed clinical data are delayed because of the need for medical chart abstractions. As of March 30, chart abstractions had been conducted for approximately 200 COVID-19 patients; the frequency and distribution of underlying conditions during this time might change as additional data become available. Clinical course and outcomes will be presented once the number of cases with complete medical chart abstractions are sufficient; many patients are still hospitalized at the time of this report. Finally, testing for SARS-CoV-2 among patients identified through COVID-NET is performed at the discretion of treating health care providers, and testing practices and capabilities might vary widely across providers and facilities. As a result, underascertainment of cases in COVID-NET is likely. Additional data on testing practices related to SARS-CoV-2 will be collected in the future to account for underascertainment using described methods (*10*).

Early data from COVID-NET suggest that COVID-19–associated hospitalizations in the United States are highest among older adults, and nearly 90% of persons hospitalized have one or more underlying medical conditions. These findings underscore the importance of preventive measures (e.g., social distancing, respiratory hygiene, and wearing face coverings in public settings where social distancing measures are difficult to maintain) to protect older adults and persons with underlying medical conditions. Ongoing monitoring of hospitalization rates, clinical characteristics, and outcomes of hospitalized patients will be important to better understand the evolving epidemiology of COVID-19 in the United States and the clinical spectrum of disease, and to help guide planning and prioritization of health care system resources.

**Summary**

**What is already known about this topic?**

Population-based rates of laboratory-confirmed coronavirus disease 2019 (COVID-19)–associated hospitalizations are lacking in the United States.

**What is added by this report?**

COVID-NET was implemented to produce robust, weekly, age-stratified COVID-19–associated hospitalization rates. Hospitalization rates increase with age and are highest among older adults; the majority of hospitalized patients have underlying conditions.

**What are the implications for public health practice?**

Strategies to prevent COVID-19, including social distancing, respiratory hygiene, and face coverings in public settings where social distancing measures are difficult to maintain, are particularly important to protect older adults and those with underlying conditions. Ongoing monitoring of hospitalization rates is critical to understanding the evolving epidemiology of COVID-19 in the United States and to guide planning and prioritization of health care resources.

## Footnotes

* These authors contributed equally.

† https://www.cdc.gov/coronavirus/2019-ncov/prevent-getting-sick/diy-cloth-face-coverings.html.

§ https://www.hhs.gov/about/agencies/iea/regional-offices/index.html.

¶ https://www.fda.gov/medical-devices/emergency-situations-medical-devices/emergency-use-authorizations.

** https://www.cdc.gov/nchs/nvss/bridged_race.htm.

†† https://www.cdc.gov/nchs/data/hestat/pregnancy/2010_pregnancy_rates.htm.
